# Development of a Linear Immobilization Carrier-Based Immunoassay for Aflatoxin

**DOI:** 10.3390/bios12050317

**Published:** 2022-05-10

**Authors:** Honglin Yan, Xiaoqian Tang, Xiaohan Liu, Yating Zheng, Minhui Zhang, Yueju Zhao, Qi Zhang

**Affiliations:** 1 Key Laboratory of Detection for Biotoxins, Key Laboratory of Biology and Genetic Improvement of Oil Crops, Ministry of Agriculture and Rural Affairs, PRC, Oil Crops Research Institute, Chinese Academy of Agricultural Sciences, Wuhan 430061, China; hlyan2019@163.com (H.Y.); tangxiaoqian@caas.cn (X.T.); liuxiaohan1103@163.com (X.L.); zhengyating227@126.com (Y.Z.); 15072595295@163.com (M.Z.); 2 Mars Global Food Safety Center, Beijing 101407, China; 3 Hubei Hongshan Laboratory, Wuhan 430000, China

**Keywords:** linear immobilization carrier, immunoassay, aflatoxin, ELISA, biosensor

## Abstract

We explored the feasibility of developing immunoassay technology with a linear carrier, to develop a simpler and cheaper rapid immunoassay technology. We selected aflatoxins as an example for research, as they are a group of highly toxic and carcinogenic compounds representing a worldwide threat to human health and life. With a non-competitive immunoassay, we detected and evaluated the effect of 28 different linear materials on antibody immobilization. Mercerized cotton and Dyneema line were chosen from the linear materials for further comparison using a competitive immunoassay, because both showed high-signal values and relatively low background noise. The results showed the sensitive IC_50_ of mercerized cotton as the reaction carrier was 0.33 ng/mL, and the linear range was 0.16~3.25 ng/mL. The sensitivity using Dyneema line as the reaction carrier was 1.16 ng/mL. The competitive curves of four sample matrices were established to evaluate the stability of the detection system; these were basically consistent with those without sample matrices. In conclusion, both mercerized cotton and Dyneema, will be suggested for the novel development of linear immobilization carrier-based immunoassays for other analytes, and especially to construct inexpensive and easy-to-obtain biological and environmental analytical technologies and biosensors.

## 1. Introduction

A point of care test (POCT) usually refers to the rapid detection that can be completed without professional technicians and equipment. In recent years, with the development of microfluidic analysis devices with a low cost and low consumption of costly material, researchers strive to find materials with a low cost and small volume for highly sensitive and rapid detection. Paper and textile have become two very important carriers of chip laboratory and sensing applications because of their low cost, wide availability and easy deformation. Recently, the proposed paper electrode and microfluidic paper-based analysis devices can be used for a high throughput, fast, stable point of care test, which makes the design of new paper-based sensors possible [1,2,3]. By constructing ordinary fibers into linear electrochemical transistor devices, it provides a way for multifunctional fabric systems [4,5,6]. A lateral flow immunochromatographic assay is a low-cost, simple, and rapid detection method, so threads have recently been proposed to transport and mix liquids in branch immunochromatographic assays [7,8,9,10,11,12].

It is difficult to prevent mycotoxins because fungi exist widely in nature. Mycotoxins are toxic low-molecular compounds, which seriously threaten the health of humans and animals. Mycotoxins are amongst the main pollutants in grain. The Food and Agriculture Organization of the United Nations reports that 25% of food production around the world is seriously contaminated with mycotoxins. Mycotoxins can destroy the growth of agricultural products, change metabolism, tissue integrity, and transcriptome response [13]. Food safety also affects international trade in food commodities. Thousands of tons of food are rejected every year because the contaminant exceeds the limit standards [14]. These toxins place a huge burden on countries that must deal with the consequences of contamination. The responses include increased public health attention, increased medical and health expenditure, and other economic tolls. According to the World Health Organization estimates for the year 2010, more than 0.4 million people died and more than 600 million became ill due to the 31 most common causes of foodborne diseases [15]. Aflatoxin (AF) is a secondary metabolite of *Aspergillus flavus* and *Aspergillus parasiticus* [16,17]. AFB_1_ mainly affects the development of rural agriculture in developing countries [18]. AFB_1_ is the most toxic and common aflatoxin [19,20], and can result in serious health problems including carcinogenesis, mutagenesis, and growth retardation [21]. AFB_1_ has genotoxicity [22] and can cause inhibited lipid utilization, defective intestinal development, and inflammation [23], and seriously threaten the health and life of human beings worldwide.

To monitor a variety of mycotoxins produced by *A. flavus*, highly sensitive and high-throughput detection methods have been developed. Chromatography is the most common commercial method for routine mycotoxin analysis in official laboratories, including thin-layer chromatography (TLC) [24], high-performance liquid chromatography (HPLC) [25,26], and liquid chromatography-tandem mass spectrometry (LC-MS) [27,28], which have made outstanding contributions in multiple mycotoxin detections. However, chromatography is limited by the need for expensive instruments, experienced operators, and cumbersome pretreatments. As a result, various portable detection technologies have been developed, and work to further develop sensitive, portable, simple, and inexpensive rapid diagnostic components is necessary.

In this project we aimed to find a microfluidic analysis device with a low cost and low consumption of costly materials. Cheap materials have been used to build sensitive, portable, simple analytical devices for the rapid detection of environmental pollution. We selected aflatoxins for this research project as they are a group of highly toxic and carcinogenic compounds that threaten human health and life worldwide, and restrict the development of national economies. We selected 28 types of polymer synthetic fiber and cotton fiber materials from the market. These shared the common morphological characteristic of being linear materials. The effects of these 28 different linear materials on antibody immobilization were detected and evaluated, and the two materials with the best immobilization effect were selected. Both materials can be used as antigen-antibody reaction carriers to complete ELISA experiments with a high sensitivity. This experiment will provide suggestions for the novel development of linear immobilization carrier-based immunoassays for other analytes, and especially to construct inexpensive and easy-to-obtain biological and environmental analytical technologies and biosensors.

## 2. Materials and Methods

### 2.1. Materials and Reagents

Aflatoxin B_1_ standards, antigens of AFB_1_, and 3,3′,5,5′-tetramethylbenzidine (TMB) were purchased from Sigma (St. Louis, MO, USA). Goat anti-mouse monoclonal antibody conjugated to horseradish peroxidase (HRP) was purchased from Solarbio (Beijing, China). The monoclonal antibody (mAb) against AFB_1_ was produced in our laboratory. Scissors, tweezers, rulers, and milk powder were purchased at a supermarket (Wuhan, China). Phosphate-buffered saline (PBS, 0.01 M, pH 7.4) was prepared by adding 8 g of NaCl, 2.9 g of Na_2_HPO_4_·12H_2_O, 0.2 g of KH_2_PO_4_, and 0.2 g of KCl to 1000 mL of deionized water. Phosphate-buffered saline with Tween-20 (PBST) was prepared by dissolving the Tween-20 with PBS buffer. Carbonate buffer solution (0.05 M, pH 9.6) was obtained by adding 2.93 g of NaHCO_3_ and 1.59 g of Na_2_CO_3_ to deionized water to make a volume of 1 L. TMB substrate chromogenic solution consists of 9.5 mL citrate buffer solution, 0.5 mL TMB solution, and 32 µL urea hydrogen peroxide solution. All other inorganic chemicals and organic solvents were of analytical reagent grade, unless stated otherwise.

Water was obtained from a Milli-Q purification system (Millipore, Danvers, MA, USA). The Coster 96-well EIA/RIA plate and 1.5 mL microtubes were purchased from Corning Incorporated (Corning, NY, USA). The absorbance at 450 nm was detected using a SpectraMax i3x Microplate Reader from Molecular Devices (Sunnyvale, CA, USA). Microplate washer was purchased form Wuhan Bai Leizhen Biotechnology Co., Ltd. (Wuhan, China).

### 2.2. Linear Material

The experiment used a variety of linear materials (Table 1). These materials were 2.5 mm ± 0.5 mm in diameter and 2 cm ± 0.5 mm in length. Monofilaments were purchased from Nantong Cintiq Monofilament Technology Co., Ltd. (Nantong, China). Other linear materials were purchased in online stores.

### 2.3. Development of Anti-AFB_1_ Monoclonal Antibody (mAb)

Cell line 1C11 screened by ELISA in our laboratory was used for cell culture to obtain monoclonal antibodies [29]. The antibody secreted by this cell line was highly sensitive to aflatoxin, so this antibody was used for the experiment.

### 2.4. Preparation of Linear Materials

Antibody proteins can bind tightly to linear materials through electrostatic, hydrophilic, and hydrophobic interactions. We used three schemes to pretreat the linear materials to improve antibody fixation. The first scheme was to maintain the original properties of these materials, rinsing them three times with ddH_2_O and drying them at 37 °C for standby. The second scheme was to use citric acid to modify the fiber materials. These materials were immersed in a mixture of 10% citric acid and 5% NaH_2_PO_4_ by fractional weight, then soaked for 5 min. After removing excessive moisture, they were pre-dried at 80 °C for 5 min, and then heated to 140 °C for 1.5 min to obtain the modified fiber materials. The third scheme was to precook these materials in boiling water containing 2 mM NaCl solution for 30 min, and then soak them in 0.01% H_2_O_2_ and 0.01 mM HCl for 5 min, in turn. The materials were fully washed with ddH_2_O and dried in an oven at 80 °C for 3 h. The best treatment method of these linear materials for antibody protein immobilization was determined by comparing these three treatment schemes.

### 2.5. Screening of Immobilizable Materials for Antibody Protein

**Figure 1:**
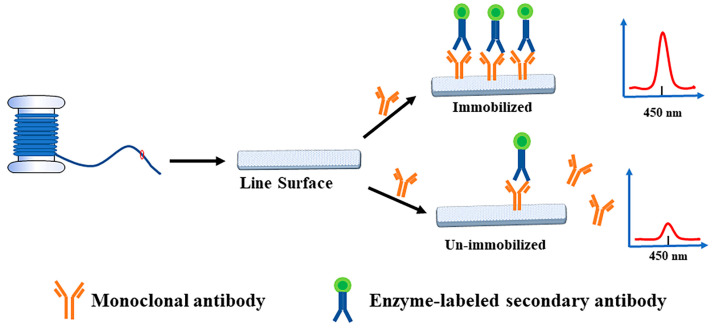
Schematic diagram for screening antibody immobilization materials.

Through the specific reaction of mAB and the enzyme-conjugated secondary antibody, the linear material that can be fixed by antibodies was screened out according to the signal changes caused by the reaction of chromogenic liquid catalyzed by enzyme-conjugated secondary antibodies, and the optimal immobilized slow-release solution of antibody protein was determined. The screening diagram of immobilized antibody materials is shown in Figure 1. The specific experimental scheme was as follows: The mAB against AFB_1_ were diluted to 1 µg/mL with PBS (pH 7.4), sodium bicarbonate (pH 8.5), and carbonate solution (pH 9.6), respectively. The linear materials were placed in an EP tube and 500 µL antibody solution was added to cover overnight at 4 °C, and the control group was established. After washing three times with the same amount of PBST solution, the unbound sites on the material were blocked with the same volume of 4% skim milk-PBST solution at 37 °C for 1 h. The washing step was then repeated. The goat anti-mouse IgG labeled with HRP diluted 1:5000 with PBST was added to the linear material and incubated at 37 °C for 1 h. After the material was washed five times with PBST, the linear material was transferred into the new EP tube by tweezers for the subsequent color reaction to exclude the influence of the original centrifuge tube on the coloration. The 200 µL TMB substrate chromogenic solution was added into the linear material and incubated at 37 °C for 10 min to make the reaction proceed. After the reaction, the color solution was fully mixed, 100 µL was removed and added to the enzyme label plate, and 50 µL concentrated sulfuric acid was added to terminate the reaction. Determination of absorbance at 450 nm by microplate reader. The optical density value reflected the immobilization of antibody proteins on the linear material, and the material with higher optical density value was selected as the best immobilization material.

### 2.6. ELISA Experiment Based on Linear Materials

To verify the application effect of linear materials in the direction of aflatoxin immunoassay technology, we subjected the screened linear materials to ELISA. The experimental scheme was adjusted for the traditional ELISA. Development of a competitive ELISA based on linear materials was as follows: Linear materials were coated with 500 µL 1 µg/mL AFB_1_-BSA overnight at 4 °C. The most suitable sustained release solution was selected for the experiment. After removal of the coating solution, 500 µL PBST was used to wash the linear material three times, and then 4% skim milk was added to seal the residual site on the material and incubated at 37 °C for 1 h. The residual milk was washed with an equal amount of PBST. To determine the optimal working concentration of AFB_1_ mAB, 500 µL of gradient diluted AFB_1_ mAB was added to the material and specifically coupled with the fixed AFB_1_-BSA. Washing steps were then repeated. A blank control was also set up by only adding PBST solution. Goat anti-mouse HRP binding antibody diluted with PBST was added to the microtubules and incubated at 37 °C for 1 h. After washing 5 times with PBST, the linear material was transferred into the new EP tube by tweezers for subsequent color reaction, and the 200 µL TMB substrate color solution was added to the linear material, and the color reaction was carried out at 37 °C for 10 min. After full oscillation, 100 µL chromogenic liquid was removed and added to the enzyme label plate, and the color reaction was terminated with concentrated sulfuric acid. The optical density was measured immediately using a high-flux microplate reader. The concentration of monoclonal antibody with optical density value of 1 was determined as the optimal working concentration.

The coating and blocking processes of linear materials were the same as the indirect non-competitive ELISA. AFB_1_ diluted with 10% methanol/PBS was added to the linear material, and mABs diluted to the optimal working concentration in equal volume were added and heated to 37 °C for 1 h to allow the reaction. The addition of secondary antibody and the coloration procedure were the same as for the noncompetitive ELISA. After the termination of coloration, the optical density (450 nm) value was measured by a high-flux microplate reader, and the standard curve of ELISA was drawn by origin plot. The sensitivity (50% inhibitory concentration) of this program was determined.

### 2.7. Matrix Effect of Line-Load ELISA

To demonstrate the versatility of this method, we purchased four types of agricultural products from a local supermarket and measured the influence of matrix effect on its sensitivity. Samples included peanut, corn, rice, and wheat, which was ground to powder by a high-speed grinder. Methanol-PBS (70:30, *v*/*v*) was added to the sample powder, and the sample was extracted by shaking at 250 rpm and 10 °C for 1 h. The extract of each sample was concentrated by centrifugation at 6000 g for 15 min. The supernatant was filtered twice using a 0.45 µm organic phase filter, and the supernatant was collected for subsequent experiments. AFB_1_ standard solution was diluted serially with matrix solution. 250 µL of diluted AFB_1_ standard solution and an equal volume of mAB diluent were added to the coated and sealed linear materials, and other conditions were the same as for the blank standard curve of AFB_1_.

### 2.8. Evaluation of Line-Load ELISA Detection Technology

Three different concentrations of AFB_1_ (0.5, 1, 2 ng/mL) were added into the blank peanut matrix and detected by the established wire-load ELISA method, and repeated detection was set for intraday (*n* = 5) and daytime (*n* = 5). The stability, reproducibility and accuracy of the detection method established with linear materials as the carrier were evaluated.

### 2.9. 96-Well Microplate Sensitivity Determination

AFB_1_ was determined with a 96-well plate as the reaction carrier. The optimum working concentration of antibody was determined. AFB_1_-BSA was diluted to 1 µg/mL with carbonate solution, coated with 100 µL AFB_1_-BSA and overnight at 4 °C. They were washed three times with PBST, then sealed in PBST with 200 µL fat-free emulsion for 2 h at 37 °C. Monoclonal antibody 1C11 was diluted with a PBST gradient, added into the wells in turn, and incubated at 37 °C for 1 h. After washing, 100 µL goat anti-mouse HRP conjugated antibody was added to the hole for 1 h. It was finally washed three times and incubated with 100 µL TMB solution for 15 min. The reaction was terminated with 2 M H_2_SO_4_ (50 µL), and the absorbance was measured at 450 nm by an enzyme-labeled instrument.

Competitive ELISA was performed on the plate. AFB_1_ was diluted in a gradient from 50 ppb, and the 12th cell was set as a blank control. The concentration of antibody with optical density value of 1 was selected as the best working concentration. 50 µL of AFB_1_ and mAb 1C11 were added into the wells. After the color reaction was terminated, the IC_50_ value of the reaction was determined.

## 3. Results and Discussion

### 3.1. Determination of Linear Material Pretreatment Scheme

**Figure 2:**
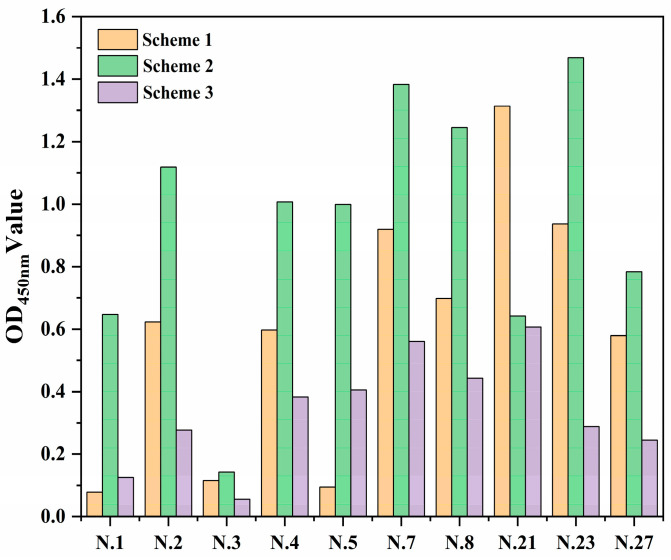
Fixation effect of antibody protein on linear materials under three different pretreatments of ddH_2_O (Scheme 1), dilute acid treatment (Scheme 2), and citric acid modification (Scheme 3).

As detailed above, we selected three different pretreatment schemes to find a better method for fixing antibody protein to the linear materials. These schemes were ddH_2_O washing (Scheme One), dilute hydrochloric acid treatment (Scheme Two), and citric acid modification (Scheme Three). We randomly selected 10 kinds of materials to determine the optimal pretreatment method. The protein immobilization effect of citric acid on modified linear materials was poor (Figure 2). This may be due to the fact that the treatment results in the development of more carboxyl structures in the linear materials and a strong negative charge in the solution, so there is a strong repulsion between the antibody protein and the linear materials. Dilute hydrochloric acid treatment enhanced the nonspecific adsorption or amide bond formation between linear materials and antibodies; thus, the linear materials had a better ability to immobilize antibody proteins after dilute hydrochloric acid treatment. Treatment with dilute hydrochloric acid was also more effective than the use of citric acid, so the second pretreatment scheme was chosen for the linear materials.

### 3.2. Material Screening to Demonstrate That mAb Can Be Immobilized

**Figure 3:**
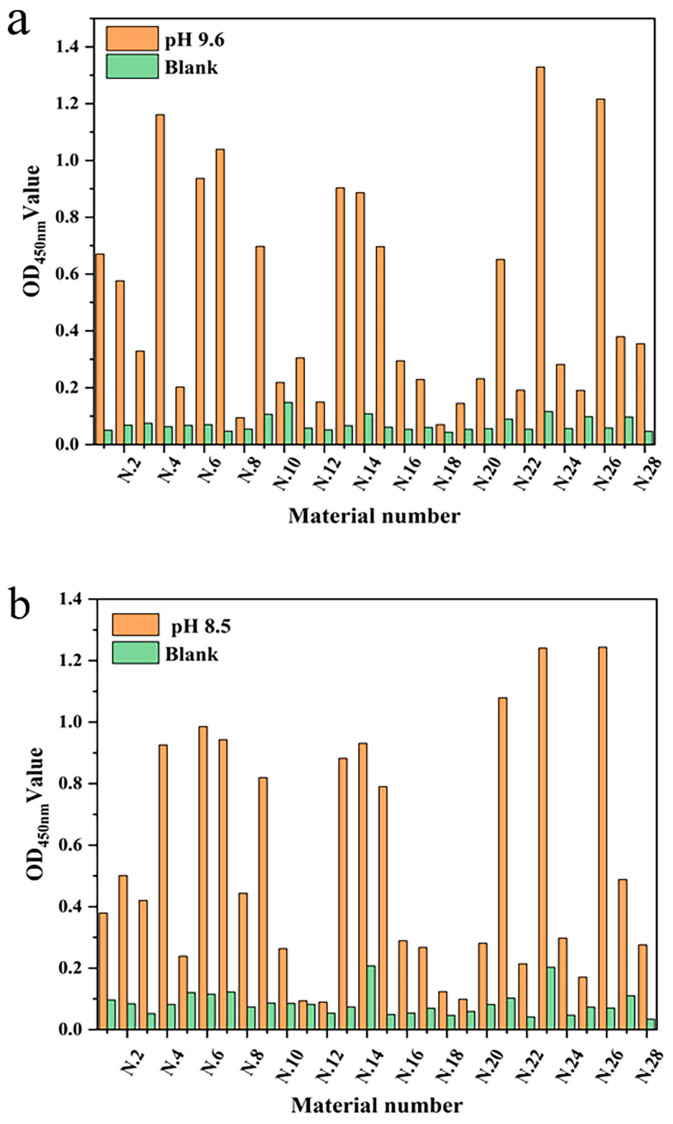

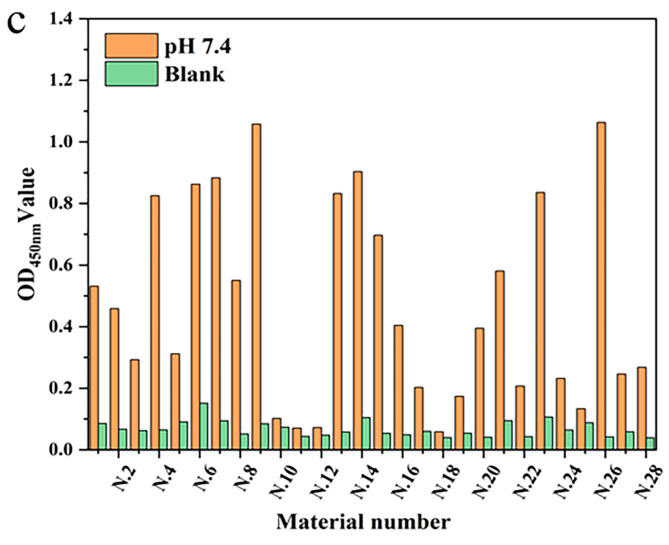
The immobilization effect of antibody protein on linear materials under different buffer conditions: (**a**) Carbonate buffer (pH 9.6); (**b**) Sodium carbonate buffer (pH 8.5); and (**c**) PBS (pH 7.4).

To verify the feasibility of mAb fixation in the linear materials, the intensities of the optical density value for different linear materials were studied under the same conditions. Twenty-eight different linear materials were selected from the market for this experiment. Three coated slow-release solutions (pH 7.4, 8.5, and 9.6) were set up to screen which of the linear materials had a better fixation of antibody protein and their optimal pH slow-release solutions. The mAb was immobilized on the linear material by non-specific adsorption and was specifically coupled with HRP-labeled signal antibody. The fixation effect of mAb was determined by measuring the optical density value (Figure 3). The results showed that each material had the ability to fix mAb, but that the fixation performance of each material was quite different. Mercerized cotton had advantages for the immobilization of mAb in carbonate solution (pH 9.6) and sodium bicarbonate buffer solution (pH 8.5), with optical density values of 1.329 and 1.240, respectively. That may have been because most of the components of mercerized cotton are cellulose, and there are high-density hydroxyl groups on the surface of cellulose. The immobilizing effect of Dyneema on mAb was second only to mercerized cotton, and showed the same performance as mercerized cotton in sodium bicarbonate solution (pH 8.5). Dyneema is a type of high-strength hydrophobic polyethylene fiber. The immobilization could be due to the salt bonds in the mAb, or that the side chains of some residues of the mAb participate in the formation of hydrogen bonds. Thus, the hydrophilicity of the residues is weakened, and the hydrophobic properties of some side chains are more prominent. Polypropylene fiber also has a good fixation effect in sodium bicarbonate solution due to this feature, but its fixation effect on antibody protein in other buffer solutions is significantly reduced. The immobilization effect of antibody protein in the alkaline solution showed advantages, which may be due to the alkaline buffer solution maintaining a higher activity of antibody protein. 

### 3.3. Establishment of Standard Curves Based on Linear Material

**Figure 4:**
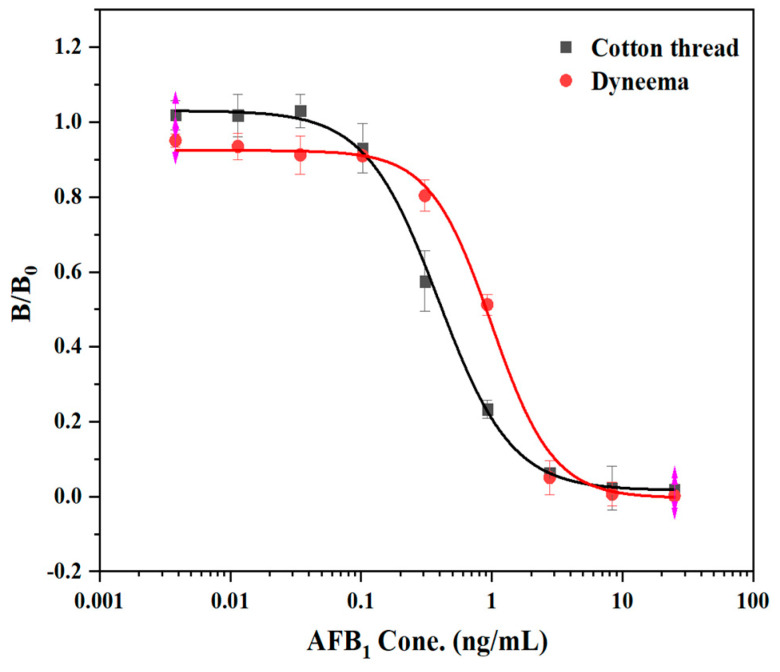
Competitive standard curve of mAb against AFB_1_ on different materials.

To determine that mercerized cotton and Dyneema are superior carriers for the immune response, we established a standard curve based on linear materials. The AFB_1_ standard was diluted with 10% methanol-PBS. The competitive inhibition curve was made with the AFB_1_ standard concentration as the X axis and B/B_0_ as the Y axis. When mercerized cotton and Dyneema were used as immune response carriers, the IC_50_’s were 0.33 ng/mL and 1.16 ng/mL, respectively (Figure 4). The former had a higher sensitivity, and the equation of the inhibition curve was y = 0.01494 + 1.00532/(1 + (x/0.307)^2.1956^), R^2^ = 0.9993. This method had a linear detection range (IC_20_–IC_80_) of 0.16–3.25 ng/mL. The sensitivity and detection linear range of this immunoreaction program were close to those of 96-well plate method. The immune response can be carried out on the intact protein fixable material.

### 3.4. Matrix Effect of Line-Load ELISA

**Figure 5:**
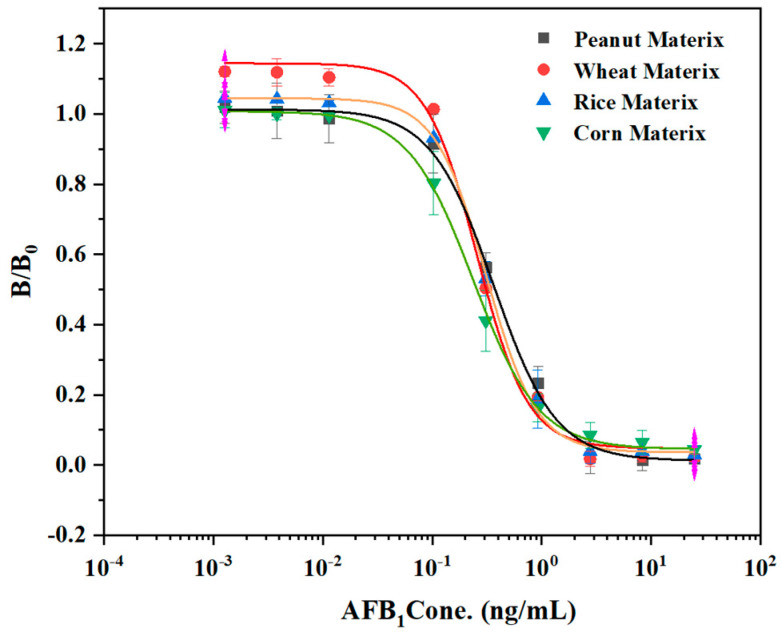
Assay standard curve of mercerized cotton in peanut, wheat, rice, and corn matrix.

The matrix effect is an important factor affecting the accuracy of ELISA. Impurities in sample extracts will cause false positive or false negative results [30]. It was found in the standard curve experiments that when mercerized cotton was used as the reaction carrier, the reaction procedure had a high sensitivity. Therefore, mercerized cotton was used to study the matrix effect on sensitivity. To explore the influence of sample matrix composition on the established method in the actual sample detection process, we purchased four agricultural products (peanut, corn, rice, and wheat) from the supermarket that were extracted with 70% methanol-PBS and prepared into a series of AFB_1_ standard solutions without dilution to establish the ELISA competition curve of each matrix extracts. The IC_50_ of peanut, corn, rice, and wheat were 0.37 ng/mL, 0.25 ng/L, 0.34 ng/mL, and 0.29 ng/mL, respectively. By comparing the IC_50_ values of the four sample matrices at a 450 nm wavelength (Figure 5), it was shown that the competition curves of the four sample matrices were not significantly different from those without a matrix. Thus, the materials used for protein immobilization can be applied to the laboratory immunoassay technology.

### 3.5. Evaluation of Line-Load ELISA Detection Technology

To evaluate the stability and reproducibility of the detection method based on linear materials, three AFB_1_ concentrations (0.5, 1, and 2 ng/mL) were added to the peanut matrix. The determination results were shown in Table 2. The recovery rate of standard addition of the line-load ELISA was between 87.88–112.88%, the intra-day coefficient of variation was 8.10–10.12%, and the inter-day coefficient of variation was 7.27–12.87%, all within the acceptable range. Therefore, the detection method based on linear materials has a certain stability and reproducibility and can be used to develop other new detection technologies.

### 3.6. Comparison of Traditional Detection Methods for AFB_1_

**Figure 6:**
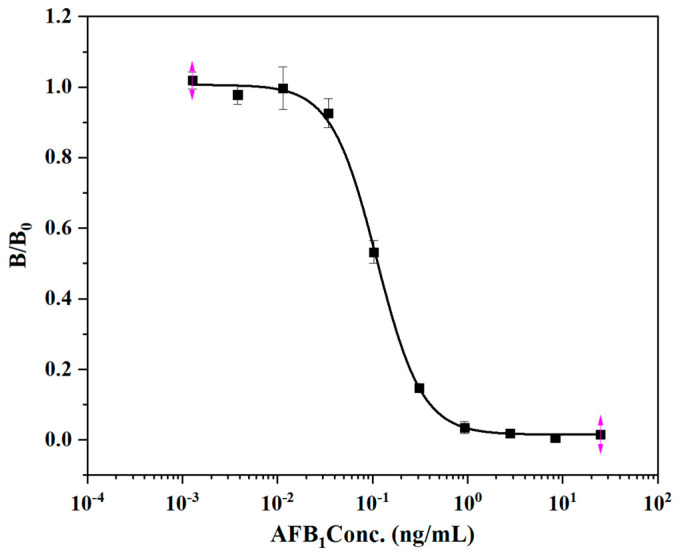
Competitive standard curve of monoclonal antibody against AFB_1_ on enzyme label plate.

The 96-well microplate was used to study the antibody protein pair for the detection of AFB_1_. AFB_1_ was diluted with 10% methanol PBS to 25, 8.3, 2.8, 0.93, 0.30, 0.10, 0.03, 0.011, 0.0038, and 0.00127 ng/mL. The mAb ELISA method based on the plate reaction was established, and the standard curve is shown below (Figure 6). The IC_50_ of this reaction is 0.114 ng/mL. There was no significant difference in the sensitivity between the established linear-ELISA detection method and the traditional 96-well enzyme-plate detection method, indicating that the rapid detection technology immobilized linear materials as the carrier to establish a new model. The traditional AFB_1_ detection method and its detection carrier components are shown in Table 3. Developing sensitive, portable, simple and cheap rapid diagnostic equipment is a technical development trend. In this experiment, the detection technology is established based on low-cost, easily available antibody immobilized linear material, and the detection cost that is much lower than traditional detection methods. It also provides carrier materials for the development of other detection technologies, and provides ideas for the development of new sensors.

## 4. Conclusions

The development of immune rapid detection technology for small molecule substances such as mycotoxins continues to be a research focus, but the component cost for rapid detection technology is high. We explored the feasibility of developing immunoassay technology using linear carriers to develop a simpler and cheaper rapid immunoassay technology. Aflatoxins were selected for our research as they are a group of highly toxic and carcinogenic compounds that threaten human health and life worldwide. The effects of 28 different linear materials on antibody immobilization were evaluated by a non-competitive immunoassay. It was shown that mercerized cotton and Dyneema wire had a stable immobilization effect. Using ELISA it was found that the IC50 of anti-AFB_1_ mAB was 0.33 ng/mL using mercerized cotton as the immune response vector. With Dyneema as the reaction carrier, the IC_50_ was 1.16 ng/mL. Mercerized cotton had a high sensitivity as the immune response carrier, and the inhibition curve equation was y = 0.01494 + 1.00532/(1 + (x/0.307)^2.1956^), R^2^ = 0.9993. The linear detection range (IC_20_–IC_80_) of this method was 0.16~3.25 ng/mL. The competition curves of the four sample matrices (peanut, corn, rice, and wheat) were not significantly different from those without matrices. The mercerized cotton and Dyneema screened in this experiment will be recommended for the development of new linear immobilized carrier immunoassays for other analytes, and to promote the development of immunoassay equipment. The antibody protein can be stably immobilized on hydrophilic and hydrophobic materials. The materials used in this experiment are cheap, easy to obtain, and have broad application prospects in the field of rapid detection, especially in the construction of semi-quantitative and quantitative analysis techniques and biosensors in biological analysis and environmental detection.

## Figures and Tables

**Table 1:** Linear materials used in the experiment.

Number	Name of Material	Number	Name of Material
N.1	Nylon monofilament	N.15	Aramid thread
N.2	Polyester monofilament	N.16	Silk thread
N.3	Acrylic monofilament	N.17	Egyptian cotton thread
N.4	Polypropylene monofilament	N.18	Steel wire
N.5	Polyphenylene sulfide fiber	N.19	Elastic line
N.6	Flat wax line	N.20	Fibrous hair
N.7	Polyvinylidene fluoride filaments	N.21	Cotton hemp thread
N.8	Polyethylene line	N.22	Aluminum steel
N.9	Reflective thread	N.23	Mercerized cotton
N.10	Rattan thread	N.24	Silk thread
N.11	Nylon thread	N.25	Cotton thread
N.12	Hemp thread	N.26	Dyneema
N.13	Conducting monofilament	N.27	EP monofilament
N.14	Cashmere thread	N.28	Polyethylene line

**Table 2:** Evaluation of line-load ELISA detection technology.

	Spiked (ng/mL)	Line-Load ELISA Mean ± SD (μg/mL)	Recovery (%)	CV (%)
Intra-assay (*n* = 5)	0.5	0.48 ± 0.04	95.88	8.32
	1	1.12 ± 0.09	112.88	8.10
	2	1.99 ± 0.20	99.64	10.12
Inter-assay (*n* = 5)	0.5	0.44 ± 0.05	87.88	11.80
	1	0.95 ± 0.12	95.26	12.87
	2	1.80 ± 0.13	90.18	7.27

**Table 3:** Carrier elements and characteristics of AFB_1_ detection method.

Detection Method	Carrier Elements	Characteristics
Thin layer chromatography (TLC)	Thin plate	Low cost; Qualitative; Semi-quantitative
High-performance liquid chromatography (HPLC)	Liquid phase	Accurate results; Complex pre-processing; Expensive instruments
Immunochromatography	NC membrane	Fast; Wide detection range; Import dependence
ELISA	Enzymatic plate	High throughput; High cost
Immunochip analysis	Electronic chip	Complex process; High cost
Biosensors	Biofilm; transducer	Complex production; High cost

## Data Availability

The data presented in this study can be obtained from the corresponding authors according to reasonable requirements.
